# Impact of seasonal hydrological variation on the distributions of tetraether lipids along the Amazon River in the central Amazon basin: implications for the MBT/CBT paleothermometer and the BIT index

**DOI:** 10.3389/fmicb.2013.00228

**Published:** 2013-08-16

**Authors:** Claudia Zell, Jung-Hyun Kim, Gwenaël Abril, Rodrigo Lima Sobrinho, Denise Dorhout, Patricia Moreira-Turcq, Jaap S. Sinninghe Damsté

**Affiliations:** ^1^Department of Marine Organic Biogeochemistry, Royal Netherlands Institute for Sea ResearchDen Burg, Netherlands; ^2^Laboratoire Environnements et Paléoenvironnements Océaniques et Continentaux, Centre National de la Recherche Scientifique, Université de BordeauxTalence, France; ^3^Department of Geochemistry, Universidade Federal FluminenseNiteroi, Rio de Janeiro, Brazil; ^4^Centre IRD France Nord, Institut de Recherche pour le Développement, UR 234GET, (ORE)-HYBAMBondy, France

**Keywords:** branched tetraethers, crenarchaeol, Amazon River, seasonal changes, MBT/CBT, BIT

## Abstract

Suspended particulate matter (SPM) was collected along the Amazon River in the central Amazon basin and in three tributaries during the rising water (RW), high water (HW), falling water (FW) and low water (LW) season. Changes in the concentration and the distribution of branched glycerol dialkyl glycerol tetraethers (brGDGTs), i.e., the methylation index of branched tetraethers (MBT) and the cyclization of brGDGTs (CBT), were seen in the Amazon main stem. The highest concentration of core lipid (CL) brGDGTs normalized to particulate organic carbon (POC) was found during the HW season. During the HW season the MBT and CBT in the Amazon main stem was also most similar to that of lowland Amazon (terra firme) soils, indicating that the highest input of soil-derived brGDGTs occurred due to increased water runoff. During the other seasons the MBT and CBT indicated an increased influence of *in situ* production of brGDGTs even though soils remained the main source of brGDGTs. Our results reveal that the influence of seasonal variation is relatively small, but can be clearly detected. Crenarchaeol was mostly produced in the river. Its concentration was lower during the HW season compared to that of the other seasons. Hence, our study shows the complexity of processes that influence the GDGT distribution during the transport from land to ocean. It emphasizes the importance of a detailed study of a river basin to interpret the MBT/CBT and BIT records for paleo reconstructions in adjacent marine setting.

## Introduction

**Figure 1:**
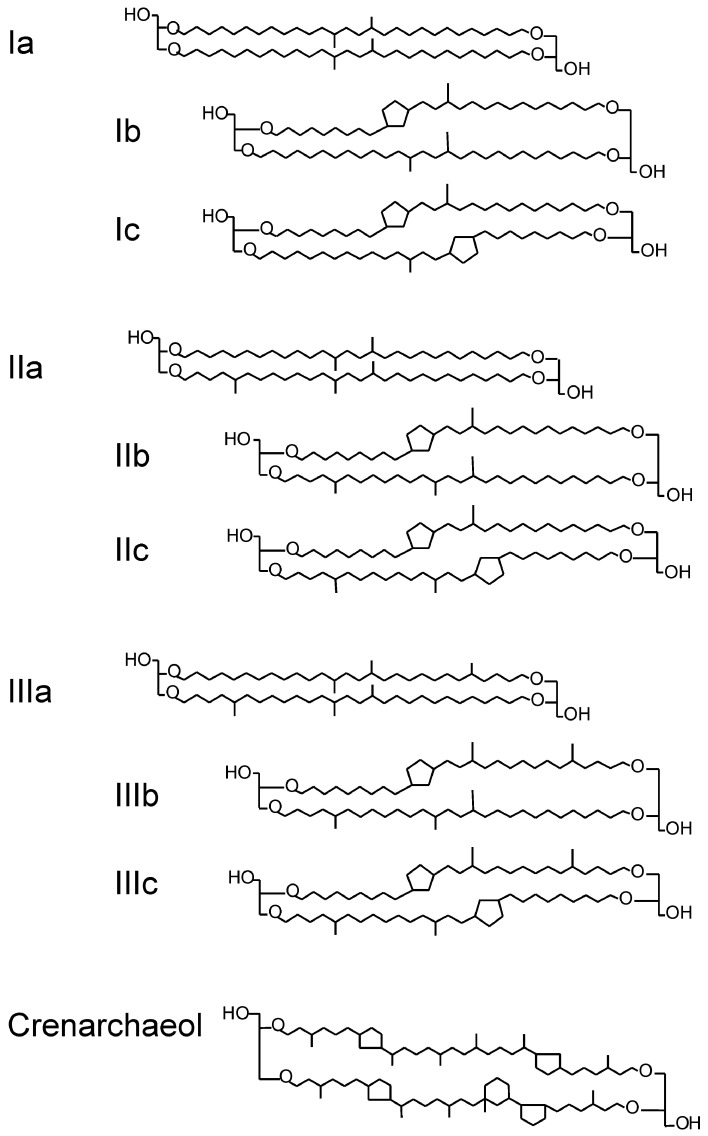
**Chemical structure of brGDGTs (Ia–IIIc) and crenarchaeol**.

Branched glycerol dialkyl glycerol tetraethers (brGDGTs) (Figure 1) are membrane-spanning lipids most likely of anaerobic (Weijers et al., 2006a,b) and heterotrophic (Pancost and Sinninghe Damsté, 2003; Oppermann et al., 2010) bacteria that are ubiquitous in peats (Weijers et al., 2006a; Huguet et al., 2010) and soils (Weijers et al., 2007a; Peterse et al., 2012). A study combining brGDGT analysis with molecular ecological techniques of Swedish peat suggested that some Acidobacterial species may produce brGDGTs (Weijers et al., 2009), which was recently confirmed by the identification of brGDGT-Ia in two acidobacterial cultures (Sinninghe Damsté et al., 2011). However, Acidobacteria so far have only been shown to produce one brGDGT; other bacterial sources of brGDGTs remain possible but are yet unidentified. Crenarchaeol (Figure 1) is a membrane-spanning lipid of Thaumarchaeota, formerly known as Group I Crenarchaeota (Brochier-Armanet et al., 2008; Spang et al., 2010). It is abundant in aquatic environments: oceans (Schouten et al., 2002; Kim et al., 2010), lakes (Blaga et al., 2009; Sinninghe Damsté et al., 2009; Powers et al., 2010), and rivers (e.g., Herfort et al., 2006; Kim et al., 2007; Zell et al., 2013), but also occurs in soils (Weijers et al., 2006b).

The brGDGTs were used to define a paleotemperature proxy (i.e., the MBT/CBT proxy), based on the fact that variations in the distributions of brGDGTs with respect to the number of methyl branches (four–six) and cyclopentane moieties (up to two) in soil (Sinninghe Damsté et al., 2000) correlated with mean annual air temperature (MAAT) and soil pH (Weijers et al., 2007a). The MBT/CBT proxy has been used to reconstruct past MAAT and soil pH changes in diverse settings: lake sediments (Tierney et al., 2010; Tyler et al., 2010; Zink et al., 2010; Fawcett et al., 2011), peat (Ballantyne et al., 2010), loess (Peterse et al., 2011), and marine sediments in front of rivers (Weijers et al., 2007b; Donders et al., 2009; Rueda et al., 2009; Bendle et al., 2010). The brGDGT and crenarchaeol concentrations are also used to calculate the branched and isoprenoid tetraether (BIT) index, which was defined as a proxy for soil organic carbon input from land to aquatic environments (Hopmans et al., 2004; Herfort et al., 2006; Kim et al., 2006; Blaga et al., 2009). It has been applied to marine and lacustrine sediment cores to reconstruct changes in river runoff and the rainfall amounts in the past (Ménot et al., 2006; Verschuren et al., 2009).

Initially, it was hypothesized that brGDGTs are mainly produced on land, transported to the ocean via rivers by soil erosion, and then deposited in marine sediments (Hopmans et al., 2004). Based on this idea, Weijers et al. (2007b) reconstructed the MAAT and the soil pH of the drainage basin of the Congo River using marine sediments deposited close to the river mouth. However, a recent study in the central Amazon basin showed that the majority of brGDGTs originates from the lowland Amazon soils, but *in situ* production in the Amazon River itself also influenced, though to a lesser extent, the brGDGT distribution (Zell et al., 2013). *In situ* production of brGDGTs was also proposed to occur in the Yangtze River (Zhu et al., 2011; Yang et al., 2013). In addition to the alteration of the brGDGT distribution, the *in situ* production of brGDGTs can influence the BIT index, which is also influenced by the crenarchaeol production in the river and in soil (Yang et al., 2013; Zell et al., 2013).

In the present study, we assessed the effects of hydrodynamical variations on the distributions and sources of brGDGTs and crenarchaeol in the central Amazon basin and their implication on the MBT/CBT proxy and the BIT index. Suspended particulate matter (SPM) samples were collected at five stations along the Amazon main stem and three tributaries (Negro, Madeira, and Tapajós) at four different hydrological seasons [rising water (RW), high water (HW), falling water (FW), and low water (LW)]. The concentration and distribution of brGDGTs of both core lipid (CL) and intact polar lipid (IPL)-derived fractions were investigated. IPL-derived fractions were applied as an indicator of GDGTs derived from more recently-living cells, since IPLs are less stable than CLs (e.g., Harvey et al., 1986).

## Study area

**Figure 2:**
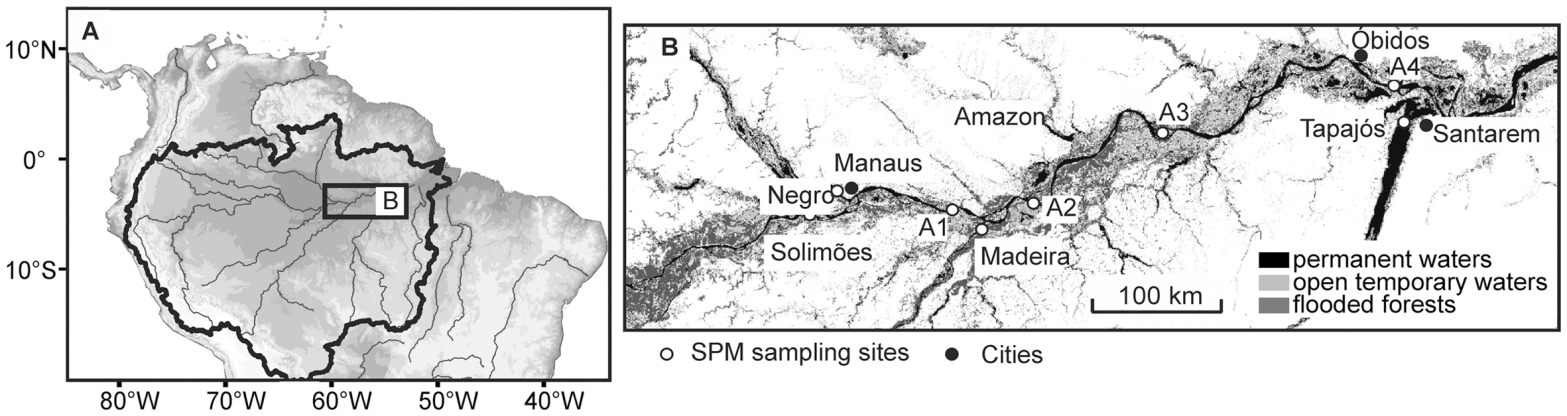
**Study area (A) showing the sampling stations along the Amazon main stem and its tributaries (B).** Note that A1–A4 indicate sampling stations Amazon 1–4.

The Amazon River is formed by the confluence of the Ucayali and Marañon Rivers in Peru and referred to as the Solimões River upstream of its confluence with the Negro River in Brazil. Our study area is located in the downstream section of the Amazon River in Brazil, from the city of Manaus on the Negro River to the city of Santarem at the confluence of the Amazon River with the Tapajós River (Figure 2). The Amazon River is the world's largest river with a drainage basin of 6.1 × 10^6^ km^2^, covering about 40% of the South American continent (Goulding et al., 2003). It has a mean annual water discharge of 2 × 10^5^ m^3^ s^−1^ (Callède et al., 2000) and an annual mean sediment discharge of 0.8–1.2 × 10^12^ kg year^−1^ (Dunne et al., 1998; Martinez et al., 2009) at Óbidos, the most downstream gauging station in the Amazon River. The Madeira River is the largest tributary of the Amazon River, which originates in the Bolivian Andes and drains the “Planalto Brasileiro” shield and the central plains, while the Negro and Tapajós Rivers originate in the lowland of the Amazon basin. Rivers within the Amazon drainage basin are traditionally classified according to their color (Sioli, 1984): Solimões/Amazon and Madeira (white), Negro (black), and Tapajós (clear).

**Figure 3:**
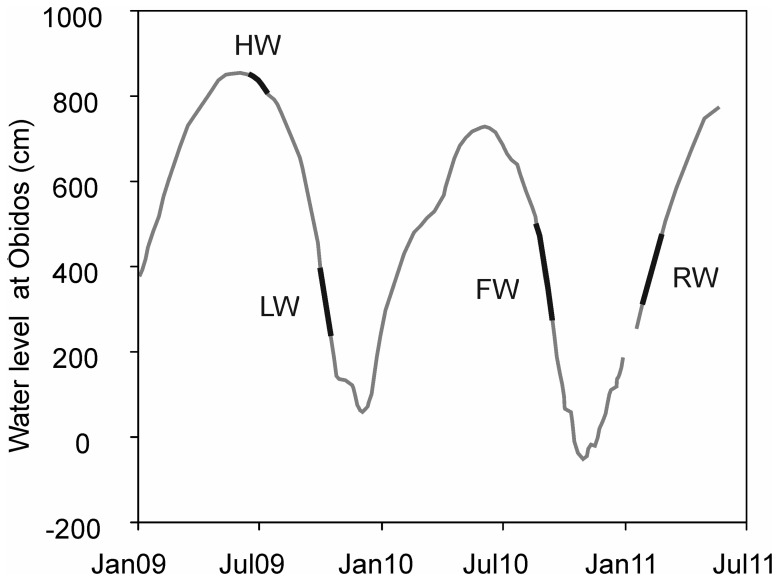
**Seasonal water level changes of the Amazon River main stem at the town Óbidos (station Amazon 4) (RW, rising water; HW, high water; FW, falling water; LW, low water)**.

Wet and dry seasons in the Amazon basin are related with fluctuations in the position of the intertropical convergence zone (Marengo et al., 2001). Precipitation ranges from <2000 mm year^−1^ in the extreme northeastern and southern parts of the basin, and increases to 7000 mm year^−1^ on the east side of the Andes (Salati et al., 1979). The Amazon River is characterized by strong water level changes between the LW (October–November) and HW (May–June) seasons (Figure 3). The water level in the Amazon main stem at Óbidos fluctuates ~10 m during an average year and its water discharge varies by a factor of 2 or 3 (Meade et al., 1979).

## Materials and methods

### Sampling

We sampled SPM at five stations along the Amazon main stem (Solimões and Amazon 1–4) and at three tributaries (Negro, Madeira, and Tapajós) (Figure 2; Table 1). SPM samples were collected in June–July 2009 (HW), in October 2009 (LW), in August–September 2010 (FW), and in January–February 2011 (RW) (Figure 3). To determine SPM concentrations, 0.5 L of water was filtered onto ashed (450°C, overnight) and pre-weighed glass-fiber filters (Whatman GF-F, 0.7 μm, 47 mm diameter). For the GDGT analysis, ~5 L of water were separately filtered onto ashed glass-fiber filters (Whatman GF-F, 0.7 μm, 142 mm diameter). The filters were kept frozen onboard and brought to the Royal Netherlands Institute for Sea Research (NIOZ, The Netherlands) laboratory, where they were freeze-dried.

**Table 1:** **Sampling stations and environmental data**.

**Sample name**	**Sampling station**	**Sampling date**	**Latitude**	**Longitude**	**Temperature (°C)**	**pH**	**SPM (mg L^−1^)**	**TOC (wt.%)**
**HIGH WATER SEASON**								
CBM 502	Solimões	20/06/2009	−3.3188	−60.5056	28	6.5	40.6	2
CBM 514	Negro	26/06/2009	−3.0746	−60.2639	29	5.0	3.1	22
CBM 516	Amazon 1	27/06/2009	−3.2412	−58.9916	28	6.4	28.6	4
CBM 517	Madeira	27/06/2009	−3.3935	−58.7775	28	6.2	53.4	2
CBM 518	Amazon 2	28/06/2009	−3.2085	−58.3050	28	6.2	27.3	4
CBM 528b	Amazon 3	01/07/2009	−2.5073	−57.3078	28	6.3	24.4	3
CBM 531	Amazon 4	03/07/2009	−1.9713	−55.4704	29	6.2	26.9	3
CBM 541b	Tapajós	07/07/2009	−2.4745	−55.0157	30	6.4	3.0	15
**LOW WATER SEASON**								
CBM 607	Solimões	05/10/2009	−3.3311	−60.5434	31	6.6	74.2	3
CBM 601	Negro	03/10/2009	−3.0727	−60.2627	31	4.7	3.8	23
CBM 620	Amazon 1	09/10/2009	−3.2423	−58.9755	31	6.4	40.2	4
CBM 621	Madeira	09/10/2009	−3.4090	−58.7852	32	7.2	33.4	2
CBM 622	Amazon 2	10/10/2009	−3.1744	−58.4091	31	6.8	22.6	3
CBM 634	Amazon 3	14/10/2009	−2.5423	−57.0267	31	6.8	31.8	3
CBM 635	Amazon 4	16/10/2009	−1.9509	−55.4904	21	6.8	31.2	3
CBM 642	Tapajós	20/10/2009	−2.5262	−55.0292	31	6.9	3.2	22
**FALLING WATER SEASON**								
CBM 706	Solimões	27/08/2010	−3.3255	−60.5528	30	6.9	37.9	2
CBM 701	Negro	25/08/2010	−3.0762	−60.2635	30	5.4	3.1	19
CBM 714	Amazon 1	01/09/2010	−3.3110	−58.8621	30	6.7	40.9	3
CBM 715	Madeira	01/09/2010	−3.4071	−58.7894	31	7.3	21.1	3
CBM 722	Amazon 2	03/09/2010	−3.1614	−58.3778	30	6.7	31.2	3
CBM 727	Amazon 3	06/09/2010	−2.5095	−57.2977	31	6.9	36.7	3
CBM 729	Amazon 4	08/09/2010	−1.9468	−55.4990	31	7.0	25.1	3
CBM 736	Tapajós	12/09/2010	−2.4578	−54.9883	31	6.8	2.7	18
**RISING WATER SEASON**								
CBM 805	Solimões	23/01/2011	−3.3254	−60.5533	29	7.1	107.5	1
CBM 801	Negro	21/01/2011	−3.0709	−60.2660	29	4.5	3.8	4
CBM 812a	Amazon 1	26/01/2011	−3.3007	−58.8720	29	7.0	130.9	2
CBM 813a	Madeira	27/01/2011	−3.4090	−58.7869	28	6.4	354.0	1
CBM 814a	Amazon 2	27/01/2011	−3.1678	−58.4211	29	6.8	149.9	1
CBM 822	Amazon 3	31/01/2011	−2.5419	−57.0026	29	6.7	127.8	1
CBM 824	Amazon 4	01/02/2011	−1.9115	−55.5535	29	6.8	114.0	1
CBM 830	Tapajós	05/02/2011	−2.5301	−55.0355	28	6.1	9.2	10

### Hydrological and environmental parameters and bulk geochemical analysis

The daily relative water level data of the Amazon River recorded at Óbidos (Figure 3) were provided by the Agência Nacional das Águas (ANA, Brazil). Water discharges at three river stations [Solimões, Madeira, and Amazon 4 (Óbidos)] were obtained from the HYBAM observatory program (http://www.ore-hybam.org/) using the site-specific discharge vs. water height relationships. The relationships were established based on the measurements with a 300 and 600 Hz Acoustic Doppler Current Profiler (ADCP, WorkHorse Rio Grande TMRD Instruments, Callède et al., 2000; Filizola and Guyot, 2004) and daily water height observations at each station. Water temperature and pH (Table 1) were measured *in situ* with a multi-parameter probe (YSI 6600 V2). Total organic carbon (TOC) content of river SPM was analyzed using an elemental analyzer C–H–N Fisions NA-2000 at the Institute for Research and Development (IRD, France) with a precision of ± 0.1 mg C g^−1^ and this was used to calculate the particulate organic carbon (POC) concentration.

### Extraction and analysis of GDGTs

Lipid extraction and analyses of CL and IPL-derived GDGTs were carried out using methods as described by Zell et al. (2013). In brief, the freeze-dried SPM filters were extracted with a modified Bligh and Dyer technique (Pitcher et al., 2009). The extracts were separated into a CL fraction and an IPL fraction on activated silica with *n*-hexane: ethyl acetate 1:1 (v:v) (CL fraction) and methanol (IPL fraction) as eluents (Oba et al., 2006; Pitcher et al., 2009). For GDGT quantification 0.1 mg C_46_ GDGT internal standard was added into each fraction (Huguet et al., 2006). Part of the IPL fraction was hydrolyzed to obtain IPL-derived CLs (Weijers et al., 2011). The CL GDGTs were analyzed using high performance liquid chromatography–atmospheric pressure positive ion chemical ionization–mass spectrometry (HPLC-APCI-MS) with an Agilent 1100 series LC/MSD SL and they were separated on an Alltech Prevail Cyano column (150 × 2.1 mm; 3 μm) using the method described by Schouten et al. (2007) and modified by Peterse et al. (2012). The compounds were eluted isocratically with 90% A and 10% B for 5 min at a flow rate of 0.2 ml min^−1^, and then with a linear gradient to 16% B for 34 min, where A = hexane and B = hexane:isopropanol 9:1 (v:v). The injection volume was 10 μl per sample. Selective ion monitoring of the [M + H]^+^ of the different brGDGTs and crenarchaeol was used to detect and quantify them. Quantification was achieved by calculating the area of its corresponding peak in the chromatogram and comparing it with the peak area of the internal standard and correcting for the different response factors (cf. Huguet et al., 2006). The analytical error was determined by duplicate measurements of 6 samples. For the concentration of the sum of brGDGTs, the analytical error was 8% for the CL brGDGTs and 6% for the IPL-derived brGDGTs. Crenarchaeol concentrations had a standard deviation of 8% (CL) and 9% (IPL-derived).

### Calculation of GDGT-based indices

The numerals refer to the GDGTs indicated in Figure 1. The BIT index (Hopmans et al., 2004), the DC (Sinninghe Damsté et al., 2009), and the MBT and CBT indices (Weijers et al., 2007b) were calculated as follows:

(1) $$\text{BIT index}=\frac{\left[\text{I}]+[\text{II}]+[\text{III}\right]}{\left[\text{I}]+[\text{II}]+[\text{III}]+[\text{IV}\right]}$$

(2) $$\text{DC}=\frac{\left[\text{Ib}]+[\text{IIb}\right]}{\left[\text{I}]+[\text{Ib}]+[\text{II}]+[\text{IIb}\right]}$$

(3) $$\text{CBT}=-\text{log}\left(\frac{\left[\text{Ib}]+[\text{IIb}\right]}{\left[\text{I}]+[\text{II}\right]}\right)$$

(4) $$\text{MBT}=\frac{\left[\text{I}]+[\text{Ib}]+[\text{Ic}\right]}{\begin{array}{l} \left[\text{I}]+[\text{Ib}]+[\text{Ic}]+[\text{II}]+[\text{IIb}\right]\\ \;+[\text{IIc}]+[\text{III}]+[\text{IIIb}]+[\text{IIIc}] \end{array}}$$

The average standard deviations of the MBT was 0.001 (CL) and 0.013 (IPL-derived), for the DC 0.001 (CL) and 0.008 (IPL-derived), for the CBT 0.007 (CL) and 0.036 (IPL-derived), and for the BIT 0.003 (CL) and 0.007 (IPL-derived).

For the calculation of pH and MAAT, the regional soil calibration for the Amazon basin (Bendle et al., 2010) was used:

(5) $$\text{CBT}=4.2313-0.5782\times \text{pH }(r^{2}=0.75)$$

(6) $$\text{MBT}=0.1874+0.0829\times \text{CBT}+0.0250\times \text{MAAT }(r^{2}=0.91)$$

### Statistical analysis

We performed the non-parametric Mann-Whitney *U*-test which does not meet the normality assumption of the One-Way analysis variance (ANOVA) to evaluate the differences in mean values between two different groups. Groups that showed significant differences (*p* < 0.05) were assigned different letters. The software SPSS 19 was used to perform the statistical tests.

## Results

### Bulk parameters: SPM and TOC

**Figure 4:**
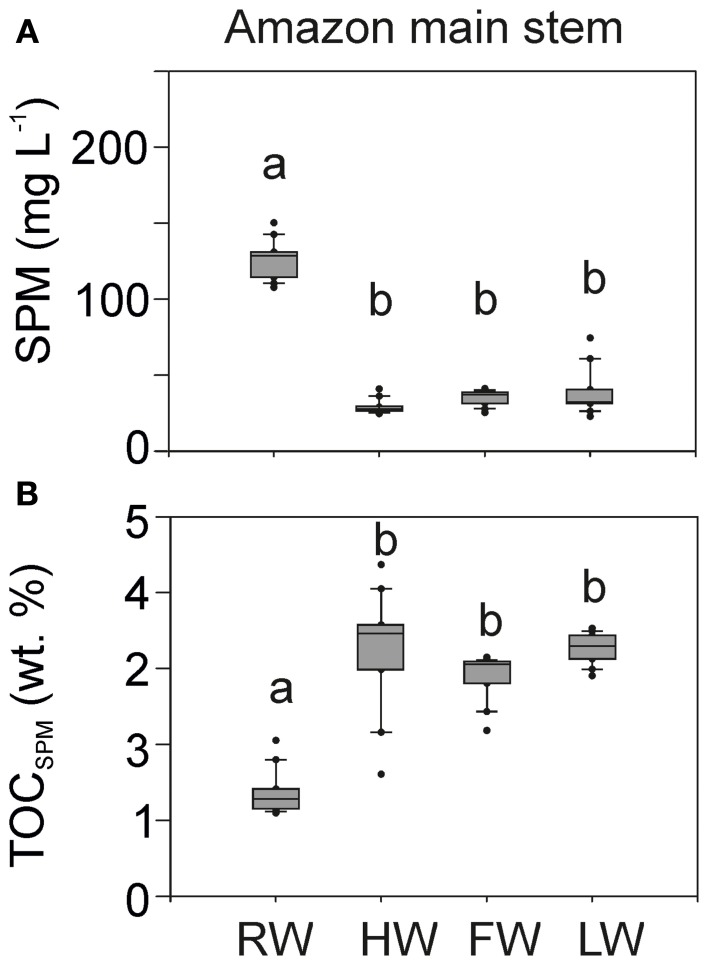
**SPM concentration (A) and TOC content of SPM in wt.% (B) in the Amazon main stem (Solimões and stations Amazon 1–4).** Letters indicate statistically significant groups of data (*p* < 0.05).

SPM concentrations varied between 20 and 150 mg L^−1^ in the Amazon main stem (Solimões and Amazon 1–4, Figure 4A). The concentrations were about three times higher during the RW season than during other seasons. The SPM concentrations in the Negro and Tapajós Rivers (on average 3 ± 0.4 and 4.5 ± 3 mg L^−1^, respectively) were low compared to those of the Madeira River (on average 115.5 ± 156 mg L^−1^) and the Amazon main stem (Table 1).

In contrast, the TOC content of the SPM in the Amazon main stem was lower during the RW season than during other seasons. Overall the TOC content of SPM in the main stem varied between 1 and 4 wt.% (Figure 4B). The TOC content of the SPM was substantially higher in the Negro and Tapajós Rivers (on average 21 and 16 wt.%, respectively) compared to the Madeira River (on average 2 wt.%) and the Amazon main stem (Table 1).

### CL and IPL-derived GDGT concentrations

**Figure 5:**
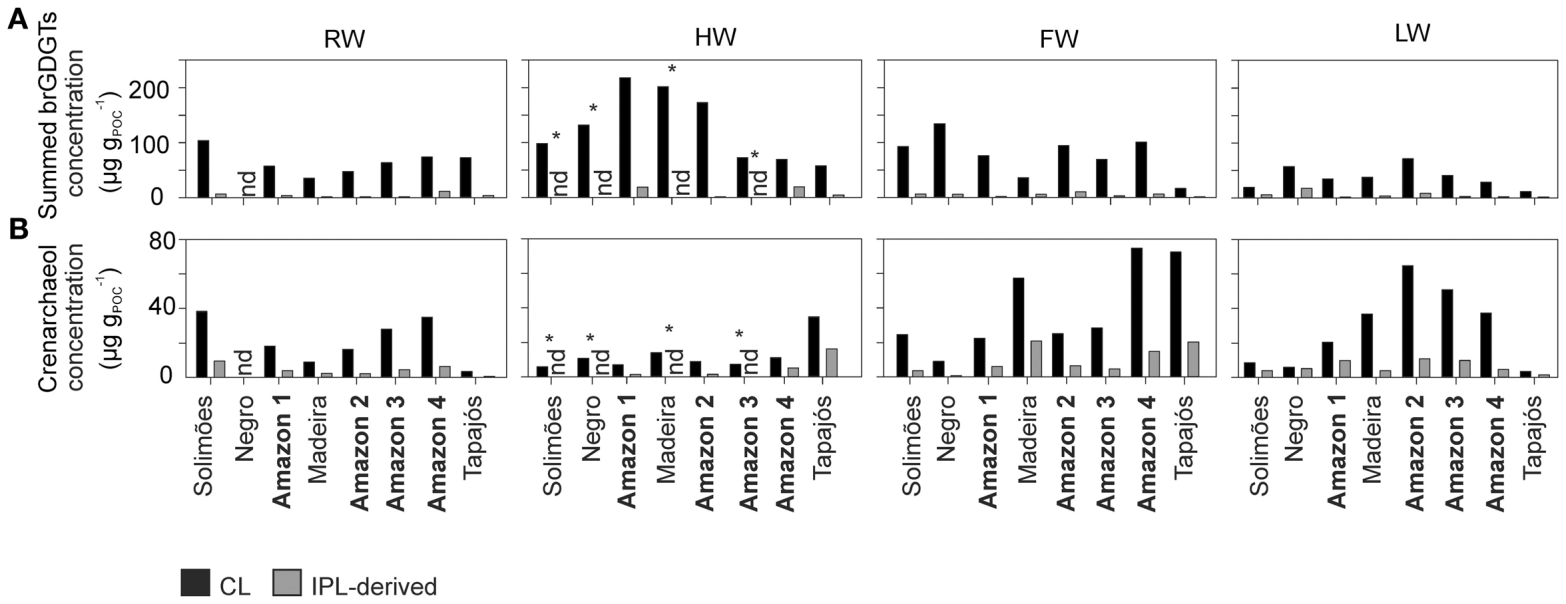
Concentration of CL and IPL-derived summed brGDGTs (A) and crenarchaeol (B) normalized to POC along the Amazon main stem and its tributaries in the four different seasons (nd, no data; ^*^ only the CL fraction was analyzed).

**Figure 6:**
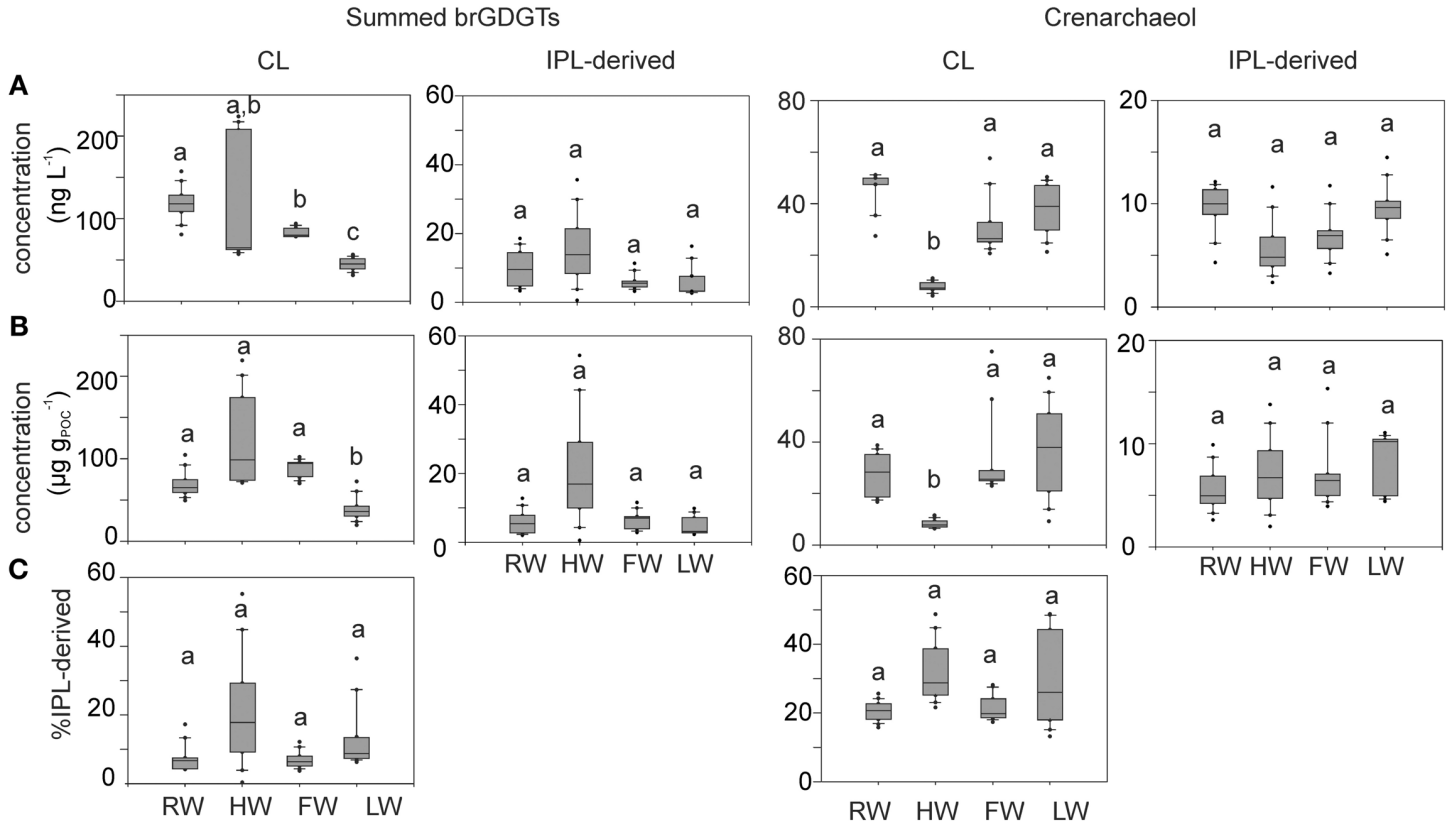
**Box plots of CL and IPL-derived summed brGDGT and crenarchaeol concentrations per liter (A) and normalized to POC (B) and the percentage of IPL-derived brGDGTs and crenarchaeol (C) in the Amazon main stem (Solimões and stations Amazon 1–4) at four different seasons.** Letters indicate statistically significant groups of data (*p* < 0.05).

Variations in the concentration of both CL and IPL-derived brGDGTs and crenarchaeol along the Amazon main stem and in the tributaries are shown in Figure 5. CL brGDGT concentrations varied between 13 and 230 μg g_POC_^−1^ (35–235 ng L^−1^) along the Amazon main stem. On average, IPL-derived brGDGTs contributed 12% to the total brGDGT pool. The CL crenarchaeol concentrations were lower than those of CL brGDGTs, ranging from 4 to 75 μg g_POC_^−1^ (5–70 ng L^−1^) along the Amazon main stem. The percentage of IPL-derived crenarchaeol was on average 36% of the total amount of crenarchaeol, with no significant difference between the seasons. However, in the HW season IPL-derived crenarchaeol could not be detected in all samples (Table 2). In order to investigate the overall hydrological effect on the concentration of brGDGTs and crenarchaeol in the central Amazon basin, the data from the stations in the Amazon main stem are illustrated in box plots, showing the concentrations in the different seasons according to the water level cycle (RW-HW-FW-LW, Figure 6). The box plots show that CL brGDGT concentrations varied over the hydrological cycle. The highest average brGDGT concentration per liter and normalized to POC occurred during the HW season, with the values of 130 ng L^−1^ and 140 μg g_POC_^−1^, but was also highly variable along the Amazon River. The lowest average concentrations (40 ng L^−1^ and 40 μg g_POC_^−1^) were found during the LW season. CL crenarchaeol concentrations showed a different pattern, with the lowest values (8 ng L^−1^ and 8 μg g_POC_^−1^) during the HW season.

**Table 2:** **GDGT concentrations**.

**Sampling site**	**CL GDGTs (μg g_POC_^−1^)**										**IPL-derived GDGTs (μg g_POC_^−1^)**									
	**Ia**	**Ib**	**Ic**	**IIa**	**IIb**	**IIc**	**IIIa**	**IIIb**	**IIIc**	**Cren.**	**Ia**	**Ib**	**Ic**	**IIa**	**IIb**	**IIc**	**IIIa**	**IIIb**	**IIIc**	**Cren.**
**HIGH WATER SEASON**																				
Solimões	81.4	5.6	1.8	15.8	2.5	–	1.3	–	–	6.3	n.d.	n.d.	n.d.	n.d.	n.d.	n.d.	n.d.	n.d.	n.d.	n.d.
Negro	120.5	2.2	0.9	8.7	0.2	–	0.1	–	–	11.1	n.d.	n.d.	n.d.	n.d.	n.d.	n.d.	n.d.	n.d.	n.d.	n.d.
Amazon 1	198.8	6.3	2.2	18.2	2.7	0.5	2.0	–	–	7.1	6.6	6.3	2.4	12.3	1.7	–	1.2	–	–	2.0
Madeira	164.6	7.8	2.8	23.6	2.8	0.2	1.4	–	–	14.2	n.d.	n.d.	n.d.	n.d.	n.d.	n.d.	n.d.	n.d.	n.d.	n.d.
Amazon 2	153.4	8.9	2.8	19.6	2.8	–	1.0	–	–	9.2	0.5	0.0	0.0	0.0	0.0	–	0.0	–	–	2.0
Amazon 3	63.2	3.3	1.1	9.9	1.1	0.2	0.6	–	–	7.7	n.d.	n.d.	n.d.	n.d.	n.d.	n.d.	n.d.	n.d.	n.d.	n.d.
Amazon 4	61.3	3.1	1.1	9.0	1.1	0.2	0.5	–	–	11.5	16.7	2.3	0.6	3.9	0.6	–	0.0	–	–	5.6
Tapajós	51.6	3.0	1.1	7.0	0.9	–	0.4	–	–	34.9	5.0	0.5	0.2	1.1	0.0	–	0.0	–	–	16.8
**LOW WATER SEASON**																				
Solimões	14.2	1.3	0.5	2.7	0.5	–	0.2	–	–	9.1	5.0	0.6	0.2	1.1	0.2	–	0.0	–	–	4.4
Negro	52.8	1.0	0.7	3.5	0.0	–	0.2	–	–	6.4	13.8	1.9	0.5	2.0	0.4	–	0.4	–	–	5.6
Amazon 1	26.7	2.2	0.9	4.7	0.8	–	0.5	–	–	20.9	1.5	0.2	0.0	0.4	0.1	–	0.0	–	–	10.2
Madeira	24.8	3.3	1.1	6.7	1.3	–	0.5	–	–	37.3	3.1	0.4	0.1	0.8	0.2	–	0.1	–	–	4.4
Amazon 2	53.5	4.8	1.8	9.8	1.8	–	0.6	–	–	64.8	7.6	0.4	0.2	1.2	0.2	–	0.2	–	–	11.0
Amazon 3	31.5	2.6	0.9	5.5	0.9	0.2	0.5	–	–	50.9	2.0	0.2	0.1	0.5	0.1	–	0.1	–	–	10.4
Amazon 4	22.1	2.2	0.8	4.1	0.7	–	0.4	–	–	37.8	1.6	0.3	0.1	0.5	0.0	–	0.1	–	–	5.0
Tapajós	9.5	1.2	0.3	1.5	0.2	–	0.2	–	–	3.9	1.9	0.3	0.2	0.5	0.1	–	0.0	–	–	1.9
**FALLING WATER SEASON**																				
Solimões	77.7	6.6	2.2	15.4	2.7	0.3	1.2	–	–	24.9	6.0	0.5	0.2	1.3	0.1	–	0.1	–	–	3.9
Negro	127.3	1.9	1.0	7.4	0.3	–	0.3	–	–	9.3	6.5	0.2	0.1	0.5	0.0	–	0.0	–	–	1.2
Amazon 1	66.2	5.1	1.8	10.6	2.0	0.2	0.5	–	–	22.9	2.1	0.2	0.1	0.7	0.1	0.2	0.0	–	–	6.4
Madeira	29.2	3.3	1.1	7.0	1.2	–	0.4	–	–	57.4	5.0	0.7	0.2	1.5	0.2	–	0.1	–	–	21.4
Amazon 2	83.5	4.9	1.8	11.2	1.8	0.3	0.6	–	–	25.5	9.7	0.7	0.3	1.8	0.3	–	0.1	–	–	7.0
Amazon 3	61.6	3.5	1.2	8.2	1.3	0.2	0.3	–	–	28.8	3.2	0.2	0.1	0.6	0.1	–	0.0	–	–	5.0
Amazon 4	87.9	5.8	2.1	13.0	2.0	0.3	1.2	–	–	75.1	5.9	0.5	0.2	1.2	0.2	–	0.1	–	–	15.3
Tapajós	15.4	2.0	0.5	2.6	0.3	–	–	–	–	72.8	1.5	0.2	0.1	0.5	0.0	–	0.0	–	–	20.7
**RISING WATER SEASON**																				
Solimões	n.d.	n.d.	n.d.	n.d.	n.d.	n.d.	n.d.	n.d.	n.d.	n.d.	n.d.	n.d.	n.d.	n.d.	n.d.	n.d.	n.d.	n.d.	n.d.	n.d.
Negro	83.3	9.9	3.3	19.5	4.5	0.6	1.9	–	–	38.7	6.0	0.6	0.2	1.6	0.3	–	0.2	–	–	9.9
Amazon 1	48.5	5.4	1.9	9.5	2.3	0.4	0.6	–	–	18.6	4.2	0.6	0.1	1.1	0.3	0.1	0.1	–	–	4.2
Madeira	28.8	3.0	1.0	7.6	1.4	0.3	0.3	–	–	9.2	1.5	0.2	0.1	0.6	0.1	–	0.0	–	–	2.7
Amazon 2	39.9	4.0	1.3	8.8	1.8	0.3	0.4	–	–	16.7	1.5	0.1	0.1	0.5	0.1	–	0.0	–	–	2.6
Amazon 3	53.2	5.2	1.8	11.2	2.4	0.4	0.5	–	–	28.2	2.0	0.1	0.1	0.5	0.1	–	0.1	–	–	5.0
Amazon 4	61.1	5.6	2.0	11.8	2.3	0.4	1.1	–	–	35.0	11.4	0.5	0.2	1.2	0.1	–	0.1	–	–	6.8
Tapajós	66.5	2.9	0.9	6.7	0.7	–	0.2	–	–	3.6	4.8	0.2	0.1	0.7	0.1	–	0.0	–	–	0.6

The tributaries (Negro, Madeira, and Tapajós) showed a similar range of CL brGDGT concentrations as those of the Amazon main stem: 60–140 μg g_POC_^−1^ (50–90 ng L^−1^), 40–200 μg g_POC_^−1^ (30–180 ng L^−1^), and 10–80 μg g_POC_^−1^ (9–69 ng L^−1^), respectively (Figure 5; Table 2). The average IPL percentage was 14% in the Negro River, 12% in the Madeira River, and 13% in the Tapajós River. The CL crenarchaeol concentrations in tributaries were 6–10 μg g_POC_^−1^ (5–8 ng L^−1^) in the Negro River, 10–60 μg g_POC_^−1^ (10–40 ng L^−1^) in the Madeira River, and 4–70 μg g_POC_^−1^ (3–35 ng L^−1^) in the Tapajós River (Figure 5; Table 2). The average IPL percentages were 35% in the Negro River, 26% in the Madeira River, and 37% in the Tapajós River.

### Distribution pattern of CL and IPL-derived GDGTs

**Figure 7:**
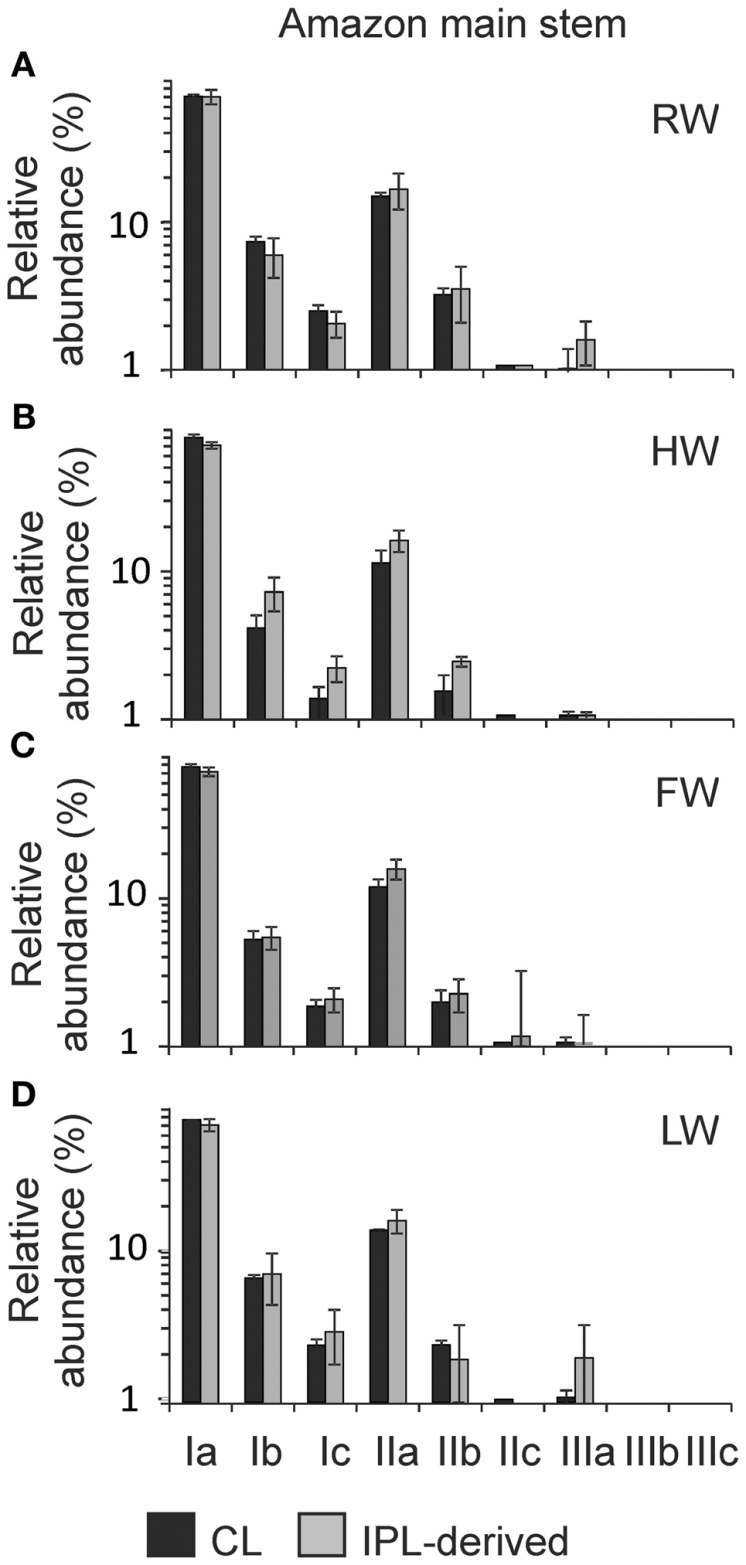
**Distribution patterns of the average relative abundances of CL and IPL-derived brGDGTs for the Amazon main stem during (A) RW, (B) HW, (C) FW, and (D) LW seasons.** Note that the y axes are logarithmic scales.

**Figure 8:**
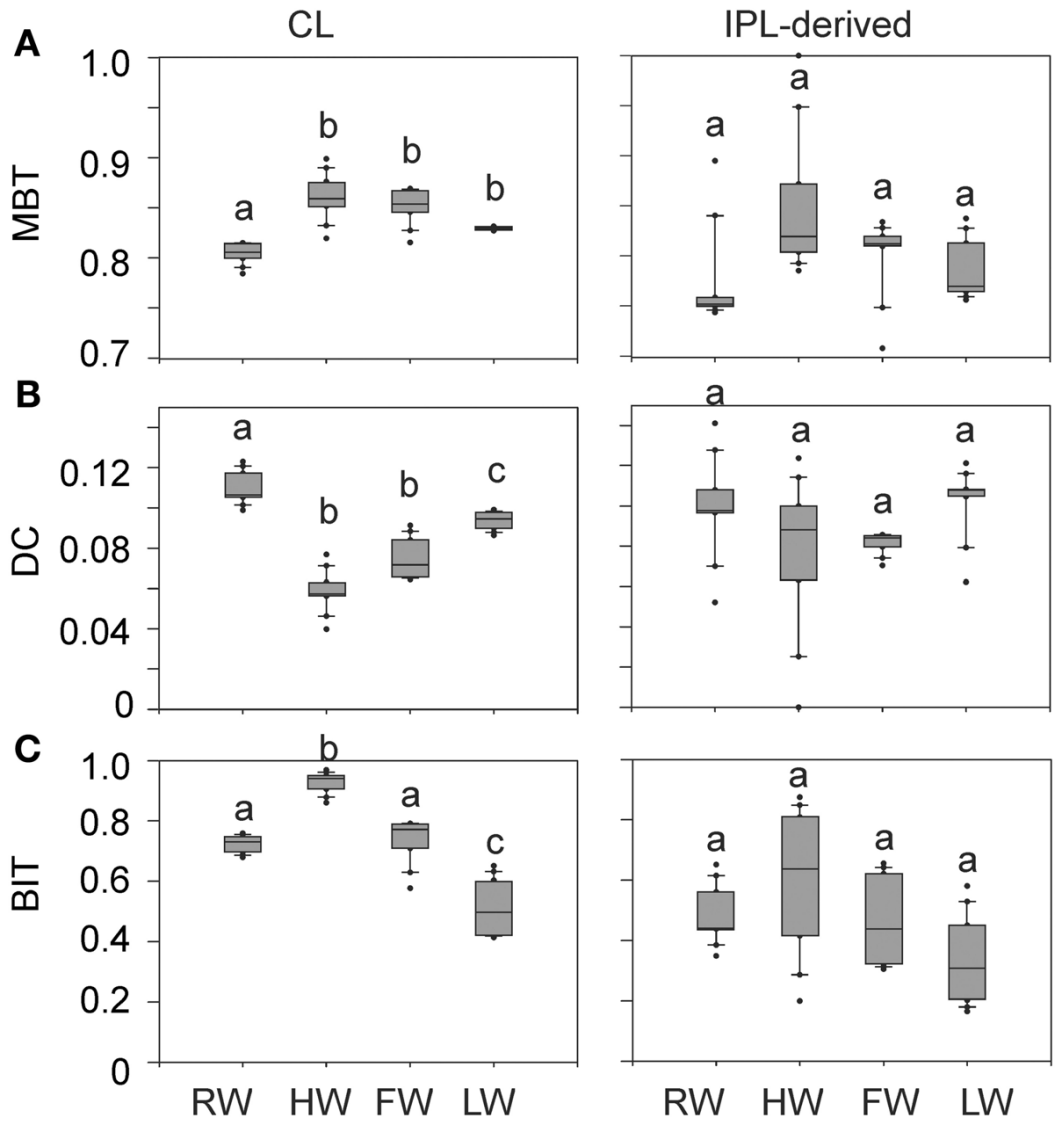
**Box plots of CL and IPL-derived MBT (A), DC (B), and BIT (C) in the Amazon main stem (Solimões and stations Amazon 1–4) at the four different seasons.** Letters indicate statistically significant groups of data (*p* < 0.05).

**Figure 9:**
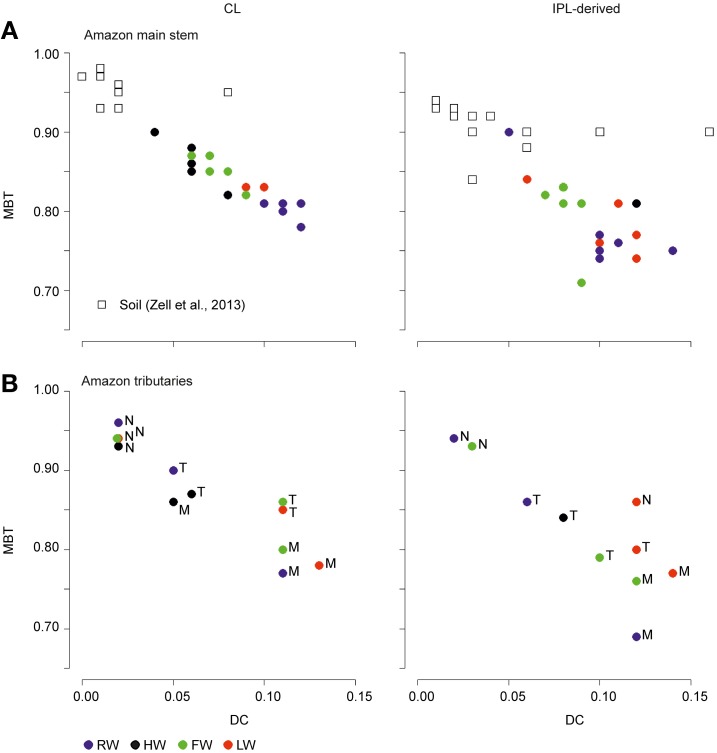
**Scatter plot of MBT vs. DC of CL and IPL-derived brGDGTs from the Amazon main stem SPM (Solimões and stations Amazon 1–4) (A) and Amazon tributaries (N, Negro; M, Madeira; T, Tapajós) (B)**.

The distribution of CL and IPL-derived brGDGTs at all stations showed a strong dominance of brGDGT Ia with a relative abundances of 70 and 80%, respectively. The second most abundant brGDGT was brGDGT IIa (Figure 7). Variations in MBT, DC, and BIT of the CLs and IPL-derived GDGTs from the Amazon main stem are illustrated in box plots, following the water level cycle (Figure 8) and scatter plots (Figure 9). In general, IPL fraction-derived indices are more variable than those of CLs. All the indices varied over the hydrological cycle. The MBT ranged from 0.78 to 0.90 for CL brGDGTs and from 0.71 to 0.90 for IPL-derived brGDGTs, with the highest average value of CL and IPL-derived brGDGTs during the HW season. The DC varied between 0.04 and 0.12 for CL brGDGTs and between 0.05 and 0.14 for the IPL-derived brGDGTs. The lowest average DC value of CL brGDGTs occurred during the HW season. The BIT ranged from 0.41 to 0.97 for CL GDGTs and from 0.16 to 0.91 for IPL-derived GDGTs. The highest average BIT value for the CL fractions (0.92) was found during the HW season, while the lowest average BIT occurred during the LW season (0.52). In the IPL-derived GDGTs a similar pattern was seen but the differences between the seasons were less clear (Figure 8C).

The MBT and DC values in tributaries were 0.77–0.96 and 0.02–0.13 for CL brGDGTs and 0.63–0.93 and 0.02–0.14 for IPL-derived brGDGTs, respectively (Figure 9B; Table 3). The CL and IPL-derived BIT values in tributaries were 0.20–0.94 and 0.09–0.94, respectively. The Negro River greatly differed from other tributaries, with higher MBT (on average 0.94) and BIT (on average 0.92) and lower DC (on average 0.02) values during all seasons (Table 3).

**Table 3:** **BIT, MBT, DC, reconstructed pH and reconstructed MAAT values of all analyzed samples**.

	**CL**					**IPL-derived**				
**Sampling site**	**BIT index**	**MBT**	**DC**	**Reconstructed pH**	**Reconstructed MAAT (°C)**	**BIT index**	**MBT**	**DC**	**Reconstructed pH**	**Reconstructed MAAT (°C)**
**HIGH WATER SEASON**										
Solimões	0.9	0.82	0.08	5.4	21.7	0.9	0.83	0.08	5.5	22.2
Negro	0.9	0.93	0.02	4.3	24.1	n.d.	n.d.	n.d.	n.d.	n.d.
Amazon 1	1.0	0.90	0.04	4.9	23.9	0.9	0.50	0.30	6.7	11.3
Madeira	0.9	0.86	0.05	5.2	22.8	n.d.	n.d.	n.d.	n.d.	n.d.
Amazon 2	0.9	0.88	0.06	5.3	23.7	0.2	n.d.	n.d.	n.d.	n.d.
Amazon 3	0.9	0.85	0.06	5.2	22.5	0.5	0.79	0.09	5.6	20.6
Amazon 4	0.9	0.86	0.06	5.2	22.8	0.8	0.81	0.12	5.8	22.1
Tapajós	0.6	0.87	0.06	5.3	23.4	0.3	0.84	0.08	5.5	22.7
**LOW WATER SEASON**										
Solimões	0.7	0.83	0.10	5.7	22.4	0.6	0.81	0.11	5.7	22.0
Negro	0.9	0.94	0.02	4.3	24.2	0.7	0.86	0.12	5.8	23.9
Amazon 1	0.6	0.83	0.09	5.6	22.3	0.2	0.76	0.10	5.7	20.0
Madeira	0.5	0.78	0.13	5.9	20.8	0.5	0.77	0.14	5.9	20.5
Amazon 2	0.5	0.83	0.09	5.6	22.5	0.4	0.84	0.06	5.3	22.1
Amazon 3	0.4	0.83	0.09	5.5	22.4	0.2	0.76	0.11	5.7	19.7
Amazon 4	0.4	0.83	0.10	5.7	22.5	0.3	0.77	0.12	5.8	20.5
Tapajós	0.7	0.85	0.11	5.8	23.6	0.6	0.80	0.12	5.8	21.7
**FALLING WATER SEASON**										
Solimões	0.8	0.82	0.09	5.6	21.8	0.7	0.81	0.08	5.5	21.5
Negro	0.9	0.94	0.02	4.2	24.3	0.9	0.93	0.03	4.7	24.7
Amazon 1	0.8	0.85	0.08	5.5	22.9	0.3	0.71	0.09	5.5	17.4
Madeira	0.4	0.80	0.11	5.8	21.4	0.2	0.76	0.12	5.9	20.1
Amazon 2	0.8	0.87	0.07	5.3	23.4	0.6	0.83	0.08	5.5	22.3
Amazon 3	0.7	0.87	0.06	5.3	23.4	0.4	0.82	0.07	5.4	21.6
Amazon 4	0.6	0.85	0.07	5.4	23.0	0.3	0.81	0.09	5.5	21.6
Tapajós	0.2	0.86	0.11	5.8	23.9	0.1	0.79	0.10	5.7	21.0
**RISING WATER SEASON**										
Solimões	0.9	0.96	0.02	4.2	24.9	0.9	0.94	0.02	4.5	24.7
Negro	0.7	0.78	0.12	5.8	21.1	0.4	0.76	0.11	5.7	19.8
Amazon 1	0.8	0.81	0.12	5.8	22.2	0.6	0.75	0.14	6.0	19.9
Madeira	0.8	0.77	0.11	5.7	20.3	0.5	0.69	0.12	5.8	17.2
Amazon 2	0.7	0.80	0.11	5.7	21.4	0.4	0.74	0.10	5.6	19.1
Amazon 3	0.7	0.81	0.11	5.7	21.6	0.3	0.75	0.10	5.6	19.4
Amazon 4	0.7	0.81	0.10	5.7	21.9	0.7	0.90	0.05	5.1	24.1
Tapajós	1.0	0.90	0.05	5.0	24.2	0.9	0.86	0.06	5.2	22.9

## Discussion

### Seasonal variation in SPM concentration and TOC of SPM

The seasonal pattern of SPM concentration in the Amazon main stem was statistically significant, with the highest concentration during the RW season (Figure 4). The SPM load is controlled by sediment erosion mostly coming from the Andes (Gibbs, 1967), but also by sediment storage and resuspension (Meade et al., 1985). Similarly, the seasonal pattern of the TOC content of SPM was statistically significant, with the lowest value during the RW season (Figure 4). Our results are in good agreement with the seasonal pattern observed at Óbidos (Amazon 4) between 1999 and 2006 (Moreira-Turcq et al., 2013).

### Seasonal variation in brGDGT concentration and source

Both CL brGDGT concentration per liter (ng L^−1^) and normalized to POC (μg g_POC_^−1^) in the Amazon main stem showed a significant seasonal pattern (Figure 6). As observed for the POC contents, the RW and LW seasons differed most significantly. In addition, in contrast to the SPM concentrations and the POC contents, significant differences in concentration (both ng L^−1^ and μg g_POC_^−1^) were also observed between the HW and LW seasons as well as between the FW and LW seasons. Interestingly, the seasonal pattern of the CL brGDGT concentrations in μg g_POC_^−1^ and to a lesser extent in ng L^−1^ was similar to the hydrological pattern of the Amazon main stem. It is noteworthy that CL brGDGT concentrations (normalized on POC) during the HW season were on average higher than in the other seasons but varied along the Amazon River. After the confluence of the tributaries Negro and Madeira Rivers with the Amazon main stem (stations Amazon 1 and 2) the concentrations were substantially higher than in the Solimões (Figure 5). This suggests that a significant amount of brGDGTs was supplied from the Negro and Madeira tributaries to the Amazon main stem.

The MBT and the DC for CL fractions in the Amazon main stem also showed clear seasonal patterns (Figures 8A,B). The differences of the CL MBT were only significant between the RW and other seasons as observed for the SPM concentrations and the organic carbon contents. Similar to the CL MBT, the differences of the CL DC were apparent between the RW and other seasons. However, the CL DC during the RW and LW season was also significantly different from those of the HW and FW seasons. The CL MBT and CL DC values of the Amazon main stem SPM during the HW season were most similar to those of lowland Amazon soils (<500 m in altitude) as well as the Negro River (Figure 9). This suggests that there was a higher input of brGDGTs derived from lowland Amazon soils during the HW season. This supports the hypothesis that soils containing brGDGTs are primarily eroded during the periods of high rainfall and surface runoff and that this material is transported by small streams and tributaries to the main river system (cf. Hopmans et al., 2004).

Our previous study of SPM sampled during the LW season (Zell et al., 2013) demonstrated that *in situ* production in the Amazon River and its tributaries (Madeira and Tapajós) might be an additional source for riverine brGDGTs, since their brGDGT distributions were substantially different from those of the lowland Amazon soils and the proportion of phospholipid-derived brGDGTs in river SPM were higher than that of the lowland Amazon soils. Our new results covering SPM from all four seasons, showing that both CL and IPL-derived MBT and DC values differed from those of the lowland Amazon soils (Figure 9), are in good agreement with the previous data. The fact that IPL-derived brGDGTs were detected in river SPM in all seasons (Figure 5) further supports the idea that *in situ* production of brGDGTs occurs in the Amazon River and its tributaries, although it should be stressed that in this study we did not analyze IPL brGDGTs directly (as performed previously; Zell et al., 2013) and that IPL brGDGTs may also partly derive from preservation of IPL brGDGTs produced *in situ* in soil. However, the %IPL content for brGDGTs in riverine SPM (11% on average) is higher than that of soils (8%; Zell et al., 2013), arguing for a contribution of riverine *in situ* produced IPL brGDGTs to the total pool of IPL brGDGTs. Variations in IPL-derived brGDGTs in the Amazon main stem over the different seasons were insignificant (Figure 6C). Overall, our data suggest that the observed variation in CL MBT and DC in the Amazon main stem (Figures 8, 9A) results from a mixing of soil-derived and *in situ* produced brGDGTs due to the variation in the contribution of brGDGT supply from the lowland Amazon soils associated with changes in precipitation and run-off and consequently soil erosion. This also holds for the tributaries (Figure 9B), except for the Negro river that is apparently dominated by soil-derived brGDGTs in all seasons.

Notably, the differences of CL MBT and CL DC in the Amazon main stem were most significant between the RW and HW seasons (Figures 8A,B, 9A). Accordingly, the CL MBT and CL DC values of SPM during the RW season were most different (i.e., lower and higher, respectively) from those of the lowland Amazon soils (Figure 9A). One possible explanation might be that the relative proportion of riverine *in situ* produced brGDGTs was largest during the RW season, while the brGDGT contribution from the lowland Amazon soils was dominant during the HW season. This could be quantitatively constrained by a two end-member mixing model, if it would be possible to constrain end-member values for MBT and DC for the *in situ* produced brGDGTs. However, the end member value of brGDGTs produced in the river could not be determined, because the IPL-derived brGDGTs in the river may still contain brGDGTs that are partly derived from soil and are transported to the river unaltered (see above). A further complication is that there might be a brGDGT source in addition to the lowland Amazon soils and riverine *in situ* production. Our previous studies (Kim et al., 2012; Zell et al., 2013) showed that erosion of high altitude (>2500 m) Andean soils had no major impact on brGDGT distributions in Amazon River SPM in the central Amazon basin. However, a small influence of Andean soils on brGDGT distributions in the lower Amazon SPM cannot be excluded. It is probable that organic carbon depleted Andean (Batjes and Dijkshoorn, 1999) soils were more strongly eroded from the Amazon and Madeira drainage basins during the RW season than other seasons which would correspond with the lower TOC content in the SPM during the RW season (Figure 4). To assess this hypothezis, more Andean soils should be investigated in future studies. On the other hand the MBT and DC in the Tapajós River, which does not receive any material from the Andes, was also different to that of soils. Therefore, we conclude that the *in situ* production of brGDGTs is the main factor that alters brGDGT distribution coming from low land soils. Interestingly a similar study by Yang et al. (2013) showed that there were no seasonal change of the MBT/CBT in the Yangtze River. This might be because there are bigger differences in the MBT/CBT along the Yangtze River and in the drainage basin soils, while the MBT/CBT along the Amazon River and in the Amazon basin soils is more constant, which makes it easier to detect seasonal differences.

### Seasonal variation in discharge of brGDGTs: implications for the MBT/CBT paleothermometer

**Figure 10:**
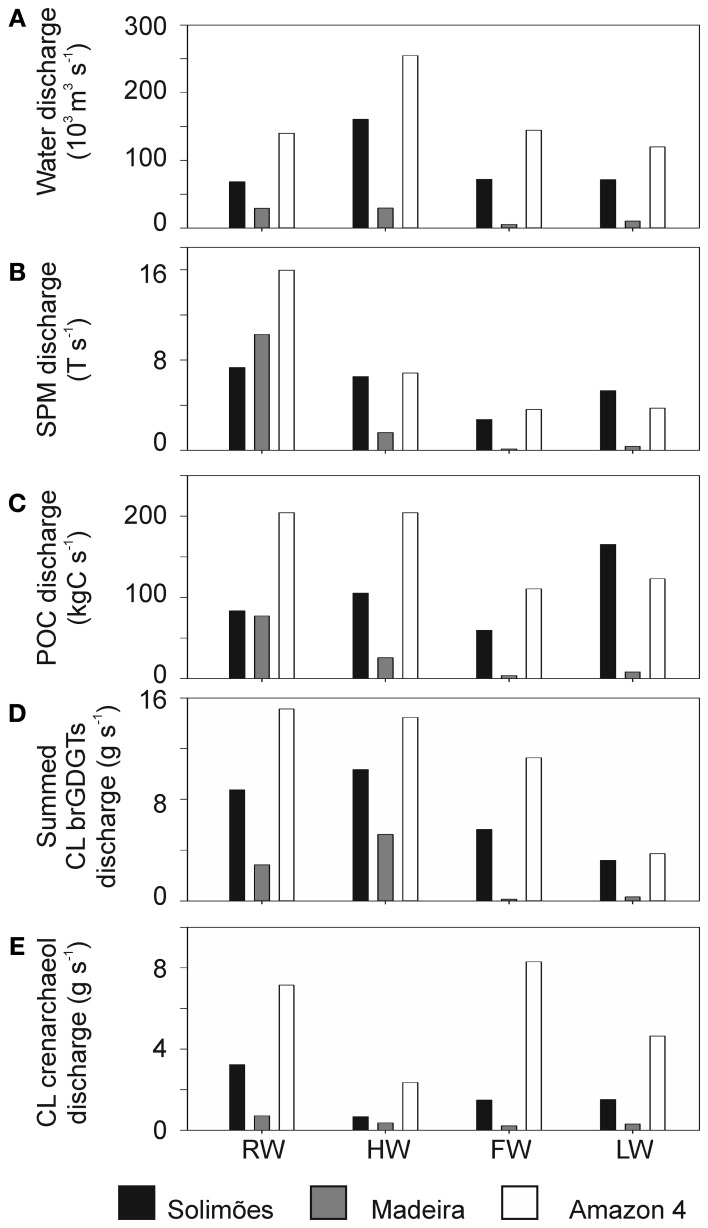
**Comparisons of (A) river water discharge (10^3^ m^3^ s^−1^) with (B) SPM discharge (T s^−1^), (C) POC discharge (kgC s^−1^), (D) CL brGDGT discharge (g s^−1^), and (E) CL crenarchaeol discharge (g s^−1^) at Solimões, Madeira and Óbidos stations**.

The water discharge along the Amazon main stem varied during the seasons between 68 and 1.1 × 10^3^ m^3^ s^−1^ at the Solimões station and between 120 and 2.5 × 10^3^ m^3^ s^−1^ at the Amazon 4 (Óbidos) station (Figure 10A). During the same periods, the water discharge varied between 5 and 30 × 10^3^ m^3^ s^−1^ at the Madeira River station. Water discharge data were not available for other river stations. To estimate the impact of the Madeira tributary to the Amazon main stem, we calculated the discharge of SPM, POC, and summed CL brGDGTs, multiplying the water discharge by the corresponding concentration at each station (Figures 10B–D). SPM discharges were highest during the RW season at all stations. Remarkably during the RW season, the SPM discharge of the Madeira exceeded that of the Solimões and contributed a large (>50%) fraction of SPM to the Amazon main stem. The POC discharge of the Madeira tributary was as high as that of the Solimões station during the RW season. However, the discharge of the summed CL brGDGTs to the Amazon main stem of the Madeira tributary was always substantially lower than that at the Solimões station. This illustrates that the importance of a tributary of a river system may vary depending on the geochemical parameter measured.

The brGDGTs discharged in each season in percent of the annual brGDGT discharge varied considerably between the stations. At the Solimões and Amazon-4 stations during the HW season, approximately one third of the annual brGDGT flux was discharged (i.e., 37 and 32%, respectively), whilst at the Madeira River station 61% of the annual brGDGT discharge occurred during the HW season. Overall, the Solimões River accounted for 50–86% of the summed CL brGDGT discharge at the Amazon 4 station and thus was the main contributor for CL brGDGTs to the Amazon River during most of the seasons assuming conservative behavior of CL brGDGTs. The contributions of the Madeira River were higher during the RW and HW seasons (19 and 36%, respectively) and much lower during the FW and LW seasons (1 and 8%, respectively). During the FW season, the contribution of the Solimões River represented only 50% of the brGDGT discharge at the Amazon 4 station. It is unlikely that the tributaries supplied all the CL brGDGTs since the brGDGT discharge in the Madeira River was low in the FW season (1%, Figure 10D). This indicates that there is an additional source of brGDGTs between the Solimões River station and the Amazon 4 station. Since water from the floodplain lakes runs into the Amazon main stem during the FW season (Bonnet et al., 2008) and also during the sampling of the LW season (cf. Figure 3), brGDGTs are carried in from the floodplain lakes and the surrounding soils. Our previous studies have already indicated that *in situ* production of brGDGTs occurs in floodplain lakes that are part of the Amazon River system in the central Amazon basin (Kim et al., 2012; Zell et al., 2013). Hence, it might be possible that floodplain lakes are an additional source of brGDGTs to the Amazon main stem during that the FW season and when the LW season was sampled. This hypothesis needs to be tested in the future.

As discussed in the previous section Seasonal Variation in brGDGT Concentration and Sources, the brGDGT distributions varied in response to the hydrological changes, which led to substantial variation in MBT and DC in the Amazon main stem (Figures 8, 9A). To assess a basin-wide integrated signal over time and space, we calculated discharge-weighted average MBT and DC values (Table 4).

**Table 4:** **Flux-weighed MBT, DC, BIT, and reconstructed pH and MAAT at Solimões, Madeira, and Amazon 4 (Óbidos) stations**.

**River station**	**GDGT fraction**	**MBT**	**DC**	**BIT index**	**Reconstructed pH**	**Reconstructed MAAT (°C)**
Solimões	CL	0.81	0.19	0.80	5.6	22
	IPL-derived	0.83	0.08	0.85	5.4	22
Madeira	CL	0.83	0.08	0.84	5.4	21
	IPL-derived	0.71	0.13	0.41	5.9	18
Amazon 4 (Óbidos)	CL	0.84	0.08	0.66	5.4	23
	IPL-derived	0.84	0.10	0.62	5.6	23

Overall, the discharge-weighted MBT values were slightly lower than those of the lowland Amazon soils for both CL and IPL-derived fractions, while the discharge-weighted DC values were slightly higher. The reconstructed pH and MAAT values calculated with the regional calibration for Amazon basin soils (Bendle et al., 2010) were 5.5–5.6 and 21.0–22.5°C for CL fractions. These estimates are different from the pH and MAAT reconstructed from MBT/CBT data of lowland Amazon soils (pH 4.4, MAAT 24°C) (Zell et al., 2013) as well as reported soil pH (~4) and MAAT (26°C) values for the lowland Amazon basin (New et al., 2002; Batjes, 2005). This demonstrates that the *in situ* production of brGDGTs affects the MBT/CBT paleothermometer in the central Amazon basin. However, compared to the influence of *in situ* production of brGDGTs in lakes, which results in much larger, differences of reconstructed temperatures compared to those of soils of the surrounding water shed (e.g., Tierney et al., 2010), the influence of *in situ* production in the Amazon River is relatively minor.

### Seasonal variation in crenarchaeol concentrations: consequences for the bit index

Our results show that river SPM contained about 40–70 times higher CL and IPL-derived crenarchaeol concentrations compared to the surrounding soils for all four seasons (crenarchaeol concentration in soil = 0.3 μg g_OC_^−1^, Zell et al., 2013). This indicates that crenarchaeol was primarily produced in the aquatic system. The presence of phospho-IPLs with crenarchaeol as CLs in the SPM samples during the LW season further confirmed the aquatic production of crenarchaeol in the Amazon River (Zell et al., 2013).

The seasonal differences of crenarchaeol concentrations in both ng L^−1^ and μg g_OC_^−1^ were only statistically significant between the HW and other seasons with substantially reduced concentrations in the HW season (Figure 6). Similar to brGDGTs, the seasonal pattern in IPL-derived crenarchaeol (%) in the Amazon main stem was statistically insignificant and varied strongly in the HW season, but was on average (28%) higher than that for brGDGTs (12%). This indicates that the production of crenarchaeol in the Amazon River is important.

The BIT index is commonly used to indicate the soil organic carbon input from soil into the ocean (Hopmans et al., 2004), but it might also reflect the soil organic carbon input to the Amazon River. The difference of the CL BIT index was significant between the HW and other seasons (Figure 8C). The IPL-derived BIT in the Amazon main stem showed similar seasonal patterns in comparison to those of the CL fractions, but these differences were insignificant due to the larger scatter. The seasonal pattern of the CL BIT index strongly resembled that of the CL brGDGT concentrations with the highest value during the HW season (Figure 6). The crenarchaeol concentration was significantly reduced during HW, which also led to a higher BIT index. Therefore, variations in the BIT index of river SPM should be interpreted cautiously: they are not only reflecting changes in soil organic matter input, but also changes in the riverine production of brGDGTs and crenarchaeol. Similar results were found in the Yangtze River which had an even bigger seasonal difference of the BIT (0.11–0.93) (Yang et al., 2013).

The CL crenarchaeol discharge (Figure 10E) was lowest during the HW season. The CL crenarchaeol input from the Solimões and Madeira Rivers only accounted for 18–45% and 3–16% of the CL crenarchaeol discharges at the Óbidos station, respectively. The discharge-weighted BIT index revealed a large difference between the Solimões station and the Amazon-4 station (Table 4). In general, the BIT index for both CL and IPL-derived fractions at the Amazon-4 station was lower than that at the Solimões station. This would all be consistent with a riverine *in situ* production of crenarchaeol as the most important. Considering that water and sediment is carried in from the floodplain lakes to the Amazon main stem (e.g., Moreira-Turcq et al., 2013), crenarchaeol produced in the floodplain lakes might also be transported to the Amazon main stem. In addition nutrient discharge from the floodplain lakes could trigger the growth of crenarchaeol in the Amazon main stem. Further investigations are thus required to better constrain how the floodplain lakes influence the crenarchaeol concentration in the Amazon River system. The decrease of the flux weighted BIT index between the Solimões and Amazon-4 station indicates that the BIT index in the Amazon River compared to soil is already lower at our study site and it might decrease even further toward the river mouth. This must be considered when the BIT index is applied in a marine sedimentary record to estimate the soil organic carbon influence from the Amazon basin.

## Conclusion

Seasonal changes in the brGDGT concentrations and the MBT and DC indicate changes of the brGDGT source. The main source is Amazon low land soil and to a smaller extent riverine *in situ* produced brGDGTs. The highest brGDGT concentrations and MBT and DC values that are most similar to those of Amazon low land soils are found during the HW season, indicating that brGDGTs from soils are washed into the river due to high rainfall and surface runoff. During the RW season the highest relative proportion of *in situ* produced brGDGTs are found. To estimate the proportion of these two sources for the final brGDGT signal, further investigation to determine the end member MBT-DC of *in situ* produced brGDGTs is needed. The difference between the MBT and DC of brGDGTs derived from soils and the flux weighted MBT and DC values from the Amazon River also leads to a small difference of the reconstructed pH and MAAT of the Amazon River (River pH 5.5–5.6, MAAT 21.0–22.5°C) and that of Amazon low land soil (pH 4.4, MAAT 24°C) (Zell et al., 2013).

Crenarchaeol is mostly produced in the river; only during HW season lower concentrations are found. Changes in both brGDGT and crenarchaeol concentrations are influencing the BIT index. Since the flux weighted BIT value is lower at the Óbidos station compared to the Solimões station, it may decrease even further toward the river mouth, which has to be considered if the BIT is used to reconstruct soil input into the marine environment.

### Conflict of interest statement

The authors declare that the research was conducted in the absence of any commercial or financial relationships that could be construed as a potential conflict of interest.
